# Online Gait Detection with an Automatic Mobile Trainer Inspired by Neuro-Developmental Treatment

**DOI:** 10.3390/s20123389

**Published:** 2020-06-15

**Authors:** Fu-Cheng Wang, You-Chi Li, Kai-Lin Wu, Po-Yin Chen, Li-Chen Fu

**Affiliations:** 1 Department of Mechanical Engineering, National Taiwan University, Taipei 10617, Taiwan; r07522829@ntu.edu.tw (Y.-C.L.); r05522801@ntu.edu.tw (K.-L.W.); 2 Department of Physical Therapy and Assistive Technology, National Yang-Ming University, Taipei 11221, Taiwan; pychen1206@ym.edu.tw; 3 Department of Computer Science & Information Engineering, National Taiwan University, Taipei 10617, Taiwan; lichen@csie.ntu.edu.tw

**Keywords:** gait training, IMU, stroke, NDT, gait detection, motor control

## Abstract

This paper demonstrates the development of an automatic mobile trainer employing inertial movement units (IMUs). The device is inspired by Neuro-Developmental Treatment (NDT), which is an effective rehabilitation method for stroke patients that promotes the relearning of motor skills by repeated training. However, traditional NDT training is very labor intensive and time consuming for therapists, thus, stroke patients usually cannot receive sufficient rehabilitation training. Therefore, we developed a mobile assisted device that can automatically repeat the therapists’ intervention and help increase patient training time. The proposed mobile trainer, which allows the users to move at their preferred speeds, consists of three systems: the gait detection system, the motor control system, and the movable mechanism. The gait detection system applies IMUs to detect the user’s gait events and triggers the motor control system accordingly. The motor control system receives the triggering signals and imitates the therapist’s intervention patterns by robust control. The movable mechanism integrates these first two systems to form a mobile gait-training device. Finally, we conducted preliminary tests and defined two performance indexes to evaluate the effectiveness of the proposed trainer. Based on the results, the mobile trainer is deemed successful at improving the testing subjects’ walking ability.

## 1. Introduction

Stroke is the second leading cause of death in the world [1], with a case occurring about every two seconds [2]. Stroke accounts for nearly 34 billion US dollars in medical costs each year in the USA [3]. The survivors usually suffer inconvenience in their daily lives and need lengthy rehabilitation to recover their abilities, such as walking by themselves. Several lower-limb rehabilitation devices have been proposed to help improve patients’ walking ability. For example, Colombo et al. [4] designed a driven gait orthosis to guide a patient’s legs on a moving treadmill. Similarly, Schmidt et al. [5] proposed the HapticWalker with programmable footplates for wheelchair-mobilized patients. Wang et al. [6] developed an active gait trainer that can actively guide the user’s ankles by motors with six bar linkages. Patton et al. [7] designed the KineAssist for gait and balance training. Esquenazi et al. [8] proposed the powered ReWalk exoskeleton that allowed the user to walk without human assistance. Schmitt et al. [9] presented the MotionMaker that had two orthoses with motors and sensors to control leg movements according to the desired positions and speeds. Bradley et al. [10] developed the NeXOS for the lower limbs of supine patients. Belforte et al. [11] designed an active gait orthosis with electro-pneumatic circuits to assist locomotion in paraplegic subjects. Yu et al. [12] proposed a compact compliant-force control actuator for portable rehabilitation robots.

Unlike these aforementioned devices, which usually guide the users to follow certain preset movements, Neuro-Developmental Treatment (NDT) is a way to let patients have the feeling of walking with minimal physical intervention [13]. Therapists need to control their patients’ joints, such as head, shoulders, and pelvis, so that patients can intentionally drive their body center of gravity (COG) forward, while balancing themselves by striding steps and feeling the COG conversion between their feet. The purpose of NDT training is to help patients to elicit positive brain reorganization and to regain control of their feet by multiple training. The gait symmetry between the healthy leg and the paretic leg can be improved during or after rehabilitation treatments by gait facilitation. However, traditional NDT training is very labor intensive and time consuming for therapists. For instance, in a clinical NDT training [14,15], one therapist stood behind the patient to correct the pelvis motion, while another therapist crouched beside the patient’s paretic limb to assist gait movements. Consequently, patients usually cannot receive sufficient training because of a lack of therapists. Therefore, Wang et al. [16] developed a stationary device that can repeat the therapists’ intervention patterns, to reduce the therapists’ working burdens and increase the patients’ training time. However, the stationary device needed to be operated in a specified space, and the patient’s forward speed was constrained by the treadmill. Therefore, this paper presents a mobile trainer that allows the subjects to walk on the ground at their preferred speeds and to receive visual feedback while walking.

The proposed mobile trainer consists of three systems: the gait detection system, the motor control system, and the movable mechanism. First, we apply IMUs to develop a gait detection system that can record the angular velocities of shanks and detect important gait events during walking. Compared with the optical motion capture systems, such as VZ4000 [17] and VICON [18], the IMU is less expensive and is portable for measuring kinematic data, allowing the extraction of gait information under different operating conditions. Second, we develop a motor control system to imitate the therapists’ interventions. We conduct traditional NDT training and record the experimental data to build an expert system that describes the therapists’ intervention patterns: stimulating the right (left) pelvis when the left (right) foot strikes the ground. Therefore, we can control the motors to recreate the therapists’ intervention patterns when the gait detection system senses heel strikes. Lastly, the gait detection system and the motor control system are integrated on a movable mechanism. We have conducted preliminary tests and defined two performance indexes to demonstrate the effectiveness of the mobile device in improving the walking ability of subjects.

The paper is arranged as follows: Section 2 introduces the mobile trainer, which is composed of a gait detection system, a motor control system, and a movable mechanism. Section 3 describes the gait detection system, which comprises two measurement units and one data logging unit. The former can estimate the gait events, while the latter sends triggering signals to the motor control system. In Section 4, we conduct clinical NDT training and record the gait data during NDT training to build an expert system that interprets the therapists’ intervention. We then design a motor control system to mimic the therapists’ intervention. Section 5 integrates the two systems and describes our clinical tests. We define two performance indexes to evaluate the performance of the proposed trainer. The results indicate that the designed trainer has positive influences on the test subjects. Finally, we draw conclusions and discuss potential future work in Section 6.

## 2. System Description

**Figure 1:**
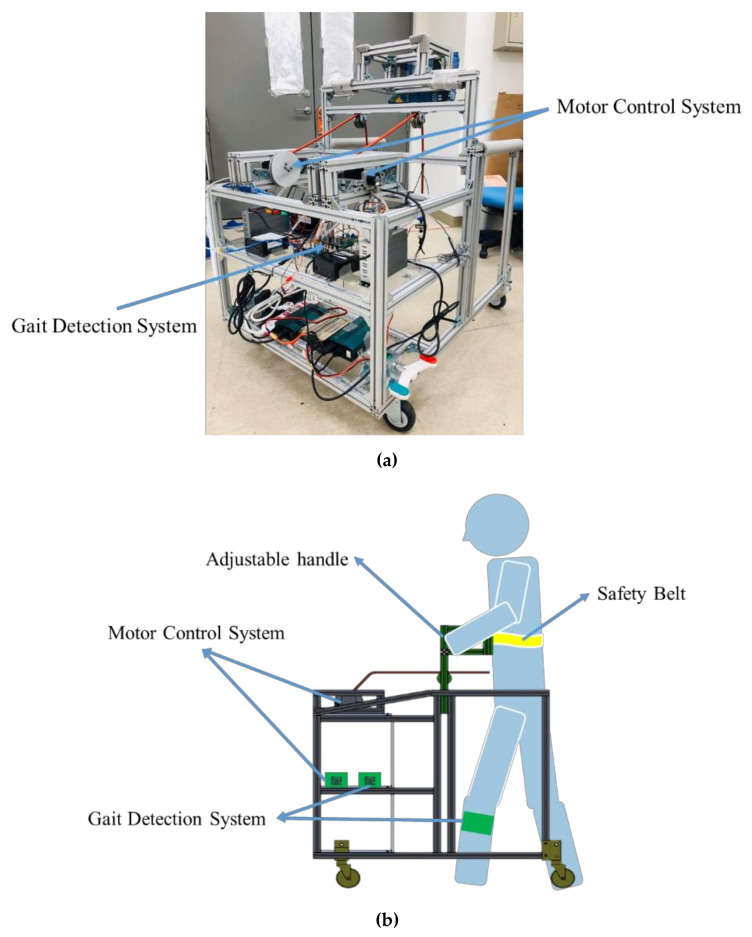

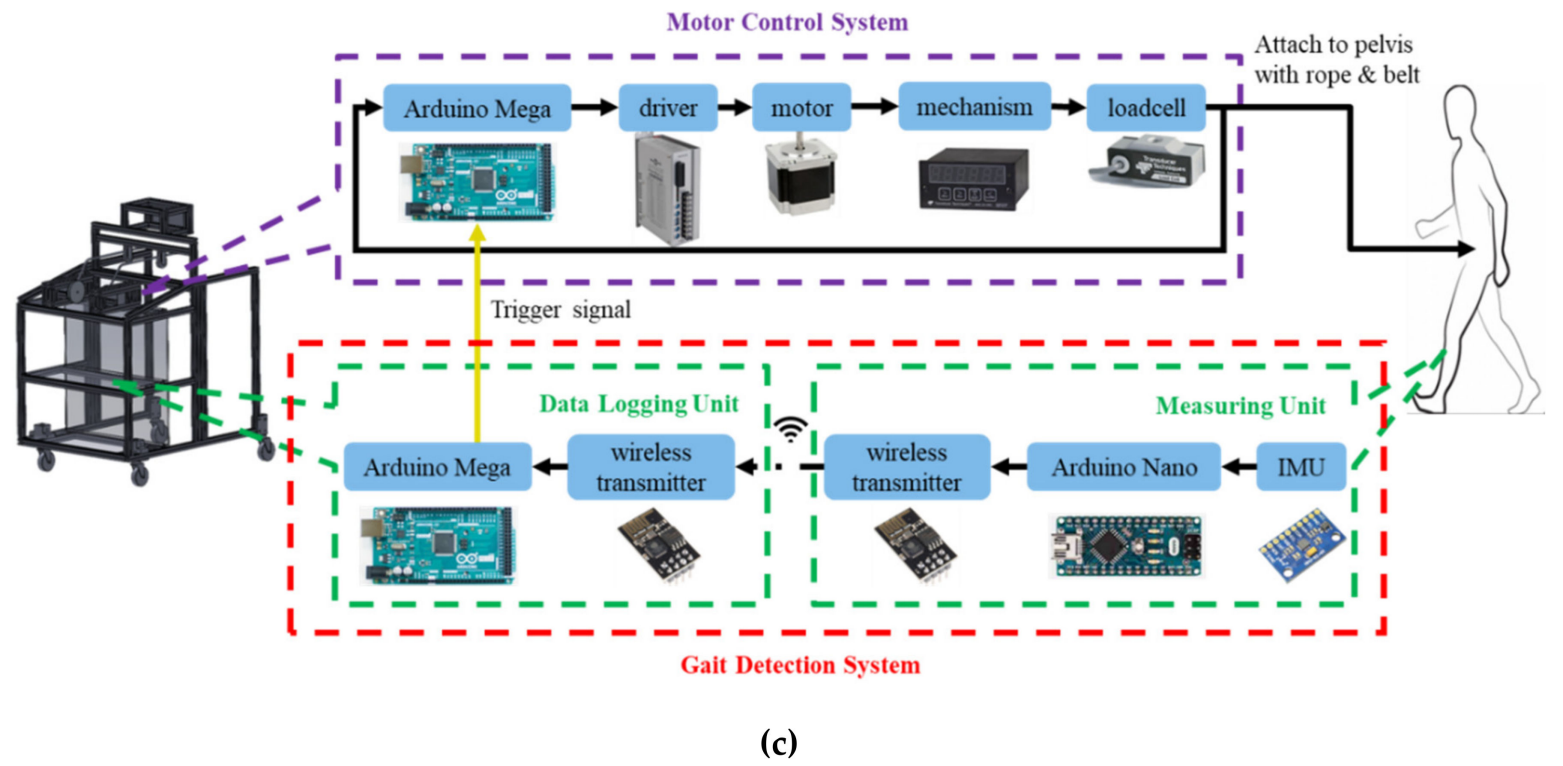
The automatic mobile trainer. (**a**) The physical structure; (**b**) the schematic diagram; (**c**) the system layout.

The mobile trainer is shown in Figure 1; it is composed of a gait detection system, a motor control system, and a movable mechanism. The gait detection system comprises two measurement units and one data logging unit. Each measurement unit is equipped with an IMU MPU-9250 [19] for measuring gaits, an Arduino Nano [20] for estimating gait events, and a wireless module ESP-01 [21] for transmitting data to the data logging unit. The data logging unit consists of a wireless module ESP-01 for receiving gait information and an Arduino Mega2560 [22] to record the gait data and to trigger the motor control system. 

The motor control system is composed of an Arduino Mega2560 to imitate the therapists’ intervention patterns, two MPK569-2.8A motors [23] to provide cuing forces through ropes, and two MLP-200 load cells [24] to measure the applied forces for feedback control. Note that we apply two Arduino Megas at the gait detection system and the motor control system so that these two systems can be operated at different sampling rates.

The movable mechanism integrates the gait detection system and the motor control system. It has four casters so that the subject can push the trainer forward when receiving training. It is also equipped with an adjustable handle and a belt for safety. The ropes connect the motors to the user’s pelvis to guide the user based on the therapists’ intervention patterns. The system specifications are illustrated in Table 1.

## 3. Gait Detection System

**Figure 2:**
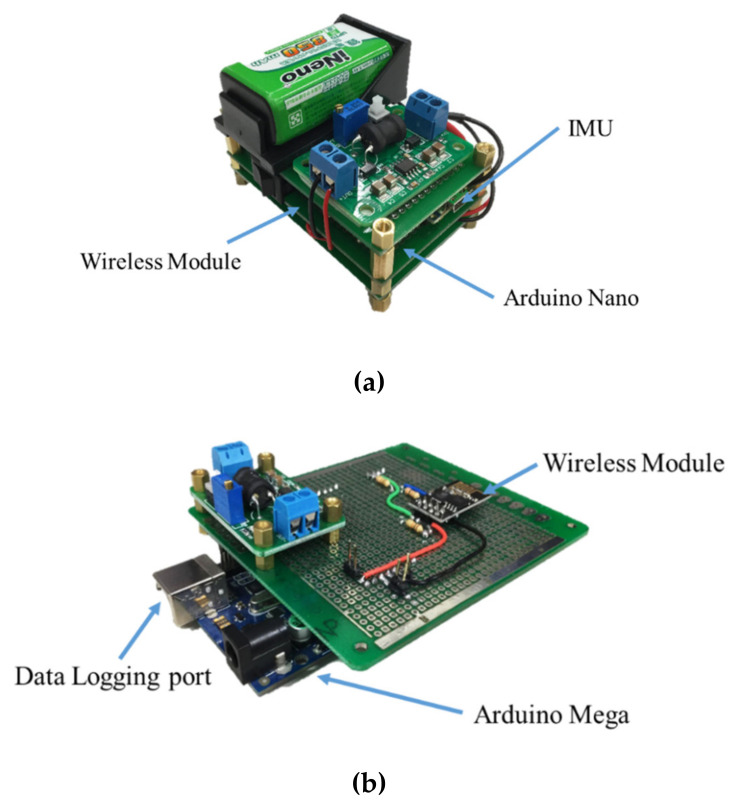
The gait detection system. (**a**) The measurement unit; (**b**) the data logging unit.

The gait detection system contains two measurement units and one data logging unit, as shown in Figure 2. The wearable measurement units are attached to the subject’s shanks to obtain the user’s kinematic data and to estimate the important gait events. The gait information is then transmitted to the data logging unit, which records the gait data and sends triggering signals to the motor control system to imitate the therapists’ intervention.

### 3.1. Gait Event Estimation

**Figure 3:**
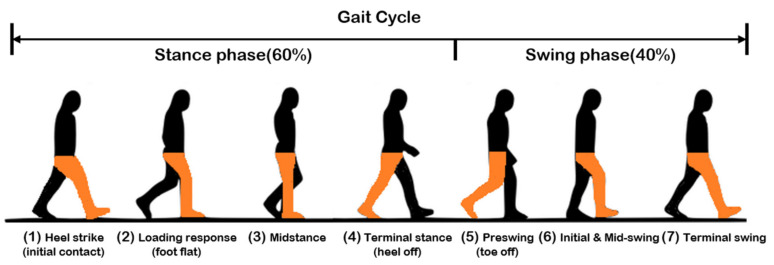
A complete gait cycle.

Human gaits are regular and periodic. A complete gait cycle is defined as a period from the heel strike (HS) to the next HS of the same leg, as shown in Figure 3 [13]. A gait cycle normally consists of about 60% stance phase and 40% swing phase, with the following three important gait events: mid-swing (MS), HS, and toe-off (TO). The gait detection system measures the gait data and estimates these gait events in real time, in order to decide the intervention timing.

**Figure 4:**
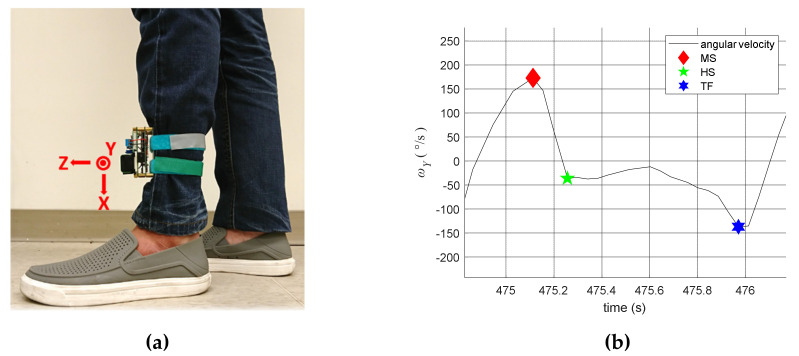
Gait measurement. (**a**) The IMU attachment; (**b**) the angular velocity.

We applied IMU to measure the angular velocity of the shank on the sagittal plane [30,31], i.e., the Y-axis in Figure 4a. The typical angular velocity responses during a complete gait phase is illustrated in Figure 4b, where the MS usually occurs with the maximum angular velocity during the gait cycle. Conversely, the HS usually happens when the angular velocity has the first negative trough after the MS, while the TO is usually associated with the negative trough before the next MS. Note that the placement of IMU sensors might affect the magnitudes of the signals because of vector projection. However, the characteristics of gait events remains the same. Therefore, we developed the following algorithms to estimate the three gait events.

1. The MS event

The MS usually accompanies the maximum angular velocity in the gait cycle. As shown in Figure 4b, we set a threshold $TH_{MS}^{\omega }=60$°/s and mark the point as the MS if its angular velocity *ω* is locally maximum and greater than this threshold, as follows:

(1) $$\omega >TH_{MS}^{\omega }$$

2. The HS event

The HS usually happens with the first negative trough after the MS, as shown in Figure 4b. Therefore, we set two thresholds, $TH_{HS}^{\omega }$ and $TH_{HS}^{t}$, to identify the HS event. The gait phase is estimated as HS if the following three conditions are satisfied:

(1) The angular velocity *ω* reaches a local minimum.
(2) The angular velocity *ω* is less than $TH_{HS}^{\omega }$, i.e., $\omega <TH_{HS}^{\omega }$.
(3) The time interval between MS and HS, labelled as $\Delta t_{HS}^{MS}$, is greater than $TH_{HS}^{\omega }$, i.e., $\Delta t_{HS}^{MS}>TH_{HS}^{t}$.

**Figure 5:**
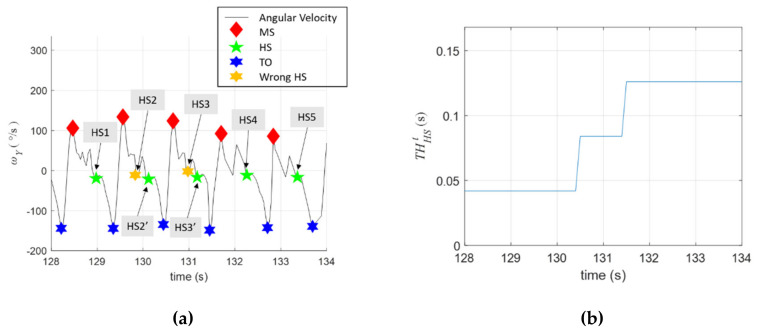
Identification of the HS events. (**a**) Labelling of the HS events; (**b**) online adjustment of $TH_{HS}^{t}$.

Referring to Figure 4b, we set $TH_{MS}^{\omega }=-5$°/s and $TH_{HS}^{t}$ with an initial value of 0.042 s, which is the sampling time of the system; that is, the HS should be at least one sample after the MS. Note that $TH_{HS}^{t}$ is adjustable because the patient’s paretic leg might have abnormal trembles and vibration during walking. The HS event can be easily identified in healthy subjects, as shown in Figure 4b. However, a stroke patient might have an uneven gait, as shown in Figure 5a, which can cause difficulties in identifying the HS event. For example, the first HS was correctly labelled as HS1, but the second HS was wrongly labelled as HS2 because a positive peak occurred afterward (i.e., the actual HS should be HS2’). Similarly, the third HS was wrongly labelled at HS3, while the correct one should be HS3’. To correct these potential errors, the threshold $TH_{HS}^{t}$ was adjusted as follows:

(2) $$TH_{HS}^{t}\leftarrow TH_{HS}^{t}+n\times T$$

where n represents the number of positive peaks after the labelled HS, while *T* is the sampling time (0.042 s). For instance, one positive peak (n=1) was evident between HS2 and the next TO, so that $TH_{HS}^{t}$ should be modified to $TH_{HS}^{t}=0.042+1\times 0.042=0.084$s. Similarly, one positive peak (n = 1) occurred between HS3 and the next TO, so that $TH_{HS}^{t}$ should be adjusted to $TH_{HS}^{t}=0.084+1\times 0.042=0.126$ sec. The online adjustment of $TH_{HS}^{t}$ is shown Figure 5b. Using the adjustment algorithm, the HS events afterward were all correctly identified. Note that $TH_{HS}^{t}$ was reduced by one sample if the HS was not successfully identified.

3. The TO event

The TO event happens after the HS and normally with the minimum angular velocity within one gait cycle. We defined two thresholds, $TH_{TO}^{\omega }$ and $TH_{TO}^{t}$, to estimate the TO events. The gait event is labelled as a TO if the following two conditions are satisfied:

(1) The angular velocity ω is less than $TH_{TO}^{\omega }$, i.e.,$\omega <TH_{TO}^{\omega }$.
(2) The time interval between HS and TO, labelled as $\Delta t_{TO}^{HS}$, is greater than $TH_{TO}^{t}$, i.e., $\Delta t_{TO}^{HS}>TH_{TO}^{t}$.

**Figure 6:**
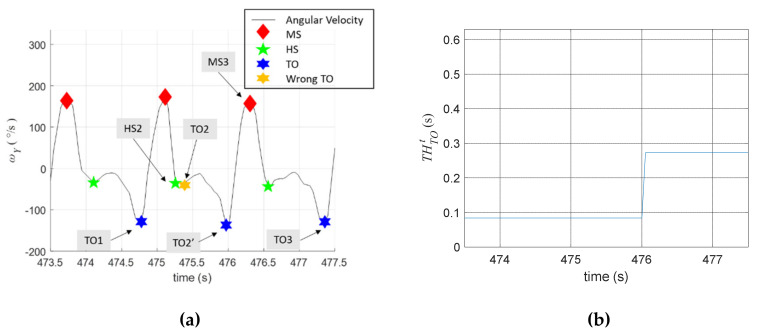
Identification of the TO events. (**a**) Labelling of the TO events; (**b**) online adjustment of $TH_{TO}^{t}$.

Referring to Figure 4b, we set $TH_{TO}^{\omega }=-35$°/s and let $TH_{TO}^{t}$ adjustable to improve the estimation accuracy. Because TO usually occurs with the last negative trough before the next MS, we adjust $TH_{TO}^{t}$ as follows:

(3) $$TH_{TO}^{t}\leftarrow TH_{TO}^{t}+\frac{1}{2}(\Delta t-\Delta t_{TO}^{HS})$$

where Δt represents the time interval between the labelled TO and the last negative trough before the next MS. The initial value of $TH_{TO}^{t}$ was set to $TH_{TO}^{t}=2\times T=0.084\;s$, i.e., the quickest TO should be at least two samples after the HS. Figure 6a shows the identification of the TO events. First, TO1 was correctly identified, but TO2 was labelled incorrectly in real time because a smaller trough (TO2’) appeared before MS3. The identification of MS3 made us realize that the correct TO should be TO2’. Because $\Delta t_{TO}^{HS}$ between HS2 and TO2 was measured as 0.126 s (three samples), while Δt between TO2 and TO2’ was measured as 0.504 s (12 samples), we adjusted $TH_{TO}^{t}$ to $TH_{TO}^{t}=0.084+(0.504-0.126)/2=0.273$. The online adjustment of $TH_{TO}^{t}$ is shown in Figure 6b. Using the adjustment algorithm, the TO events afterward were all correctly identified.

The system also calculated the average stride time of the previous three gait cycles and set it as an upper limit for $TH_{HS}^{t}\;\mathrm{and}\;TH_{TO}^{t}$.

### 3.2. Implementation and Tests

**Figure 7:**
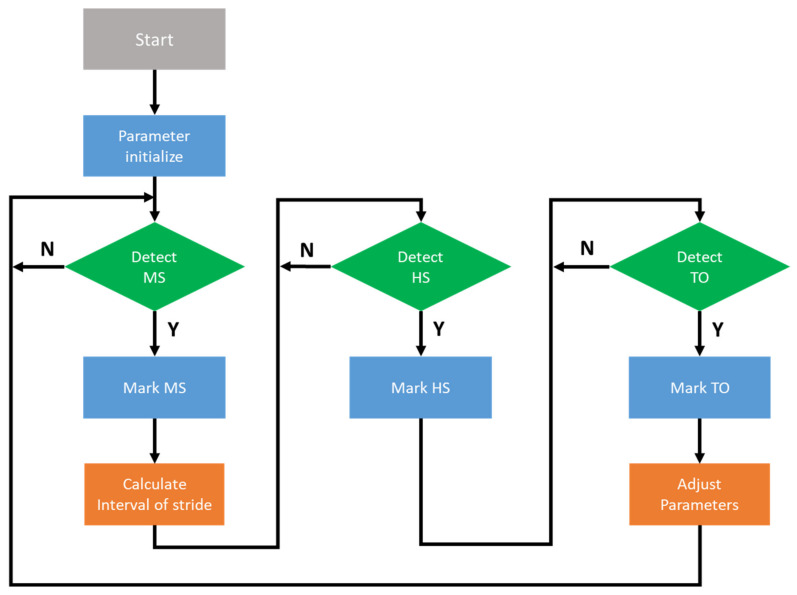
Flow chart of the gait detection algorithms.

The gait detection system applies these algorithms to detect the three gait events (MS, HS, and TO) sequentially, as shown in Figure 7.

**Figure 8:**
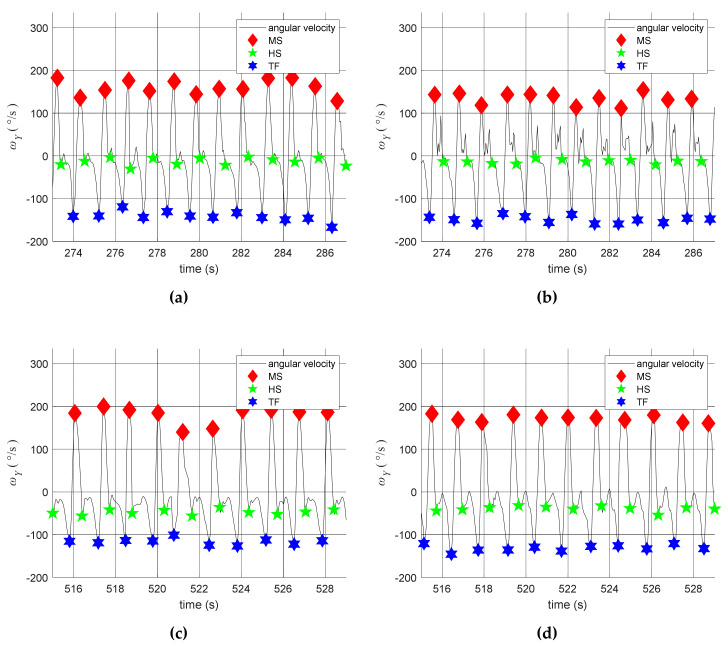
The detected gait events. (**a**) On the healthy leg of P1; (**b**) on the paretic leg of P1; (**c**) on the healthy leg of P2; (**d**) on the paretic leg of P2.

**Figure 9:**
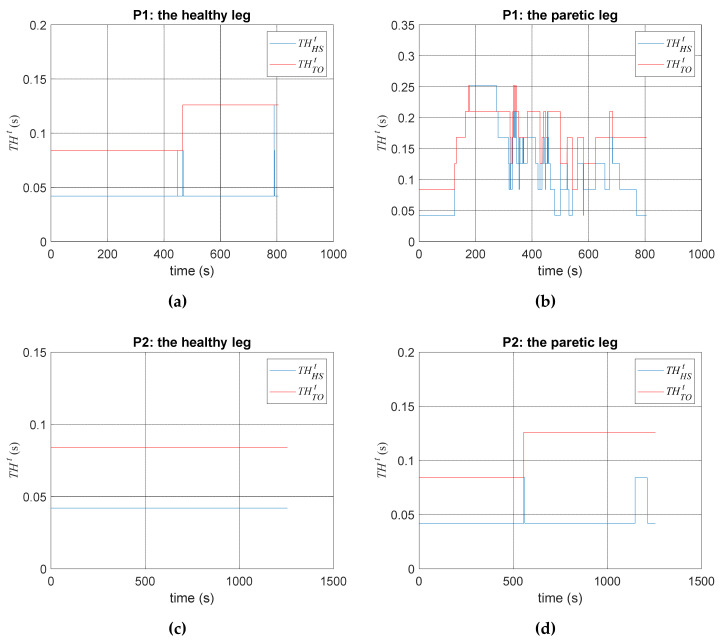
Parameter adjustment for gait detection. (**a**) On the healthy leg of P1; (**b**) on the paretic leg of P1; (**c**) on the healthy leg of P2; (**d**) on the paretic leg of P2.

We invited two stroke patients to participate in experiments. Their information is shown in Table 2. Each patient walked about 600 steps in 12 min. The testing results are shown in Figure 8. First, the gait of the healthy legs, as shown in Figure 8a,c, were regular and easy to identify. By contrast, the gait of the paretic legs contained certain noises and vibration, as shown in Figure 8b,d. Second, using our detection algorithms, the detection system could correctly identify both subjects’ gait events during walking. Third, the automatic adjustments of $TH_{HS}^{t}\;\mathrm{and}\;TH_{TO}^{t}$ are shown in Figure 9, where the parameters on the paretic side were adjusted more frequently than on the healthy side because the paretic legs tended to have abnormal tremble and vibration during walking.

We checked the effectiveness of the gait detection system by comparing its results with a VZ4000 motion capture system [18]. The successful rate of the gait detection system is defined as follows:

(4) $$P_{success}=\frac{N_{step\text{-}detected}}{N_{step\text{-}total}}\times 100\%$$

where $N_{step\text{-}total}$ is the total steps obtained by the VZ4000 motion capture system, while $N_{step\text{-}detected}$ is the number of detected HS by the proposed detection system in real time. We emphasized the detection of the HS because the automatic trainer begins the intervention upon detecting HS, as described in [16]. The walking patterns and gait parameters varied significantly in individuals. The proposed algorithms can automatically adjust the parameters, as shown in Figure 9. We set the initial values of these parameters based on the experimental data (see Figure 4b), and applied the algorithms to make real-time adjustment of the parameters for individual users. Based on this automatic adjustment, the successful rates are shown in Table 3, where the gait detection system achieved a successful rate of more than 95%. That is, it can correctly identify the HS events for triggering the motor system to repeat the therapists’ intervention, as introduced in Section 4.

## 4. Motor Control System

The trainer is designed to repeat the therapists’ intervention using ropes. As observed in [16], the therapists applied forces on the anterior superior iliac spine (ASIS) when they perceived the patients’ HS on the opposite sides. Therefore, the motor control system was designed to act as follows: pulling the right (left) ASIS when detecting the left (right) HS. In addition, the intervention force patterns were approximated as sinusoidal signals [30]. Therefore, we can control the motors to track the following force commands when detecting the HS:

(5) $$F(t)=\frac{\left(F_{\max}-F_{\min}\right)}{2}\times \sin(2\pi ft)+\frac{\left(F_{\max}+F_{\min}\right)}{2}$$

where $F_{\max}$ and $F_{\min}$, respectively, represent the maximum and minimum applied forces and f is the intervention frequency. Table 4 illustrates the parameter settings for P2. Note that these parameters can be varied for different individuals.

**Figure 10:**
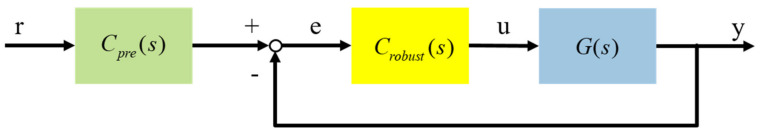
Block diagram of the motor control system.

The block diagram of the motor control system is shown in Figure 10, where G(s) represents the motor system. The identification and control design for the motors are illustrated in Appendix A. We repeated the identification experiments ten times, while considering system variation and human disturbances during NDT training. We then applied the gap metric to select the following nominal plant for the control design:

(6) $$G(s)=\frac{-16.95s+351.9}{s^{2}+38.9s+114.7}$$

The robust loop-shaping techniques were applied to design the following controller, as illustrated in Appendix A, to control the motors for imitating the therapists’ intervention patterns:

(7) $$C(s)=\frac{1.671s+10.5}{0.03183s^{2}+s}\cdot \frac{-2.801s^{3}-201.5s^{2}-3991s-14390}{s^{3}+103.9s^{2}+7049s+40320}$$

We then designed the following pre-compensator $C_{pre}(s)$ to amend the phase lag:

(8) $$C_{pre}(s)=\frac{0.143s+0.4}{0.0709s+1}$$

**Figure 11:**
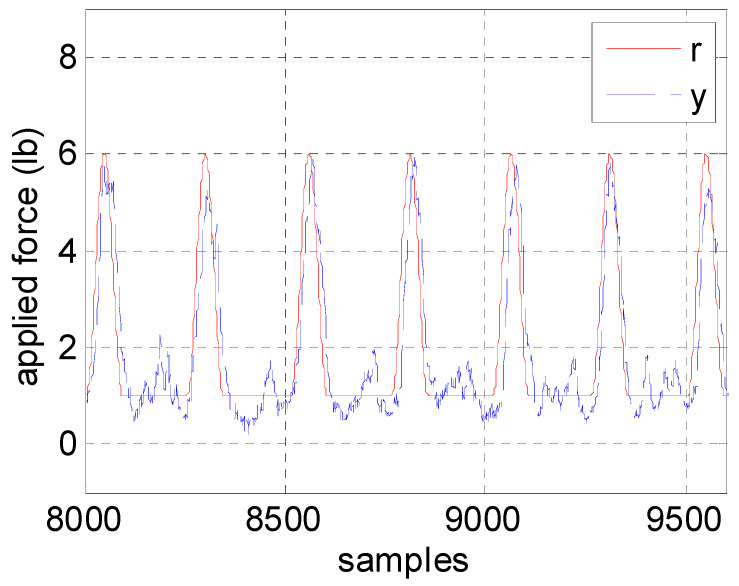
Force responses employing the motor control system.

Before conducting clinical experiments, we tested the motor control system by attaching the ropes to a subject. The detection system sent triggering signals to the motor control system when it detected the HS events. The motors, when receiving the triggering signals, began to track the sinusoidal commands then followed a minimum force of 1 lb to keep the ropes straight. Note that the motors would follow a force command of 1 lb if the system failed to detect the HS events. This small force would not harm the users. The testing results are shown in Figure 11, where the motors successfully followed the force commands with a RMSE of 0.6464 lb. That is, the designed robust control can effectively repeat the therapists’ intervention patterns even when user disturbances were introduced during NDT training. Therefore, we invited five subjects to participate in experiments, as shown in the next section.

## 5. Experimental Results

We invited five subjects, including three healthy subjects and two stroke patients, to test the mobile trainer. The information of these subjects is illustrated in Table 2 and Table 5. All subjects were informed of the purpose of the study and signed the informed consent document approved by Human Subject Research Ethics Committee of Institutional Review Board (IRB) [32] before participating. A rehab gaiter was applied to the healthy subjects to limit the joint movement of one knee, so that they would imitate the stroke gaits and receive training by the trainer. The stroke patients were selected based on the following criteria: (1) a Brunnstrom Stage (BS) [33] of 3–5; (2) a Functional Ambulation Category (FAC) [34] of 3–5; (3) ability to walk for more than 10 min with or without aid devices; (4) ability to stand up by themselves with a handrail or other aids; and (5) a Mini-Mental State Examination (MMSE) [35] score higher than 24.

Each subject received the tests by the following $A\text{-}B\text{-}\bar{A}$ procedures, where A, B, and $\bar{A}$ represent before treatment, during treatment, and after treatment, respectively. The subjects first walked by themselves for about 3 min (A), then received NDT training by the trainer for about 6 min (B), and finally walked by themselves for about 3 min ($\bar{A}$). We recorded their gait data to analyze the effects of the trainer intervention.

Because gait symmetry is critical for post-stroke walking rehabilitation, we defined the following two performance indexes to evaluate the effectiveness of rehabilitation:

(1) Ratio of the swing time [36]: The ratio of the swing time is defined as follows [36]:
  (9) $$R_{SW}=\frac{T_{SW}(P)}{T_{SW}(\mathrm{NP})}$$
  where $T_{SW}(P)$ and $T_{SW}(\mathrm{NP})$ represent the swing time on the paretic and non-paretic side, respectively. The swing time is defined as a time interval from TO to the next HS on the same side. The swing time of two legs is usually symmetrical for healthy persons, but tends to be uneven for stroke patients because of hemiparalysis. Therefore, the rehabilitation training is said to be effective if the swing time is more symmetric, i.e., $R_{SW}$ is closer to one [37].
(2) Asymmetry of the swing phase [38]: The asymmetry of the swing phase is defined as follows:
  (10) $$Asym_{SP}=\frac{P_{SP}(P)-P_{SP}(\mathrm{NP})}{P_{SP}(P)}\times 100\%$$
  where $P_{SP}(P)$ and $P_{SP}(\mathrm{NP})$ represent the proportion of the swing phase on the paretic side and the non-paretic side, respectively. The proportion of the swing phase is defined as:
  (11) $$P_{SP}=\frac{T_{SW}}{T_{gait}}$$
  in which $T_{gait}$ is the duration of one complete gait cycle, while $T_{SW}$ is the swing time of that gait cycle. For healthy persons, their gaits are usually symmetric and the swing time takes about 40% of the complete gaits on both sides. By contrast, stroke patients tend to have a deviation on the paretic side because of hemiparalysis. Therefore, we can use $Asym_{SP}$ to evaluate the impacts of the training. The rehabilitation training is said to be effective if $Asym_{SP}$ approaches zero.

**Figure 12:**
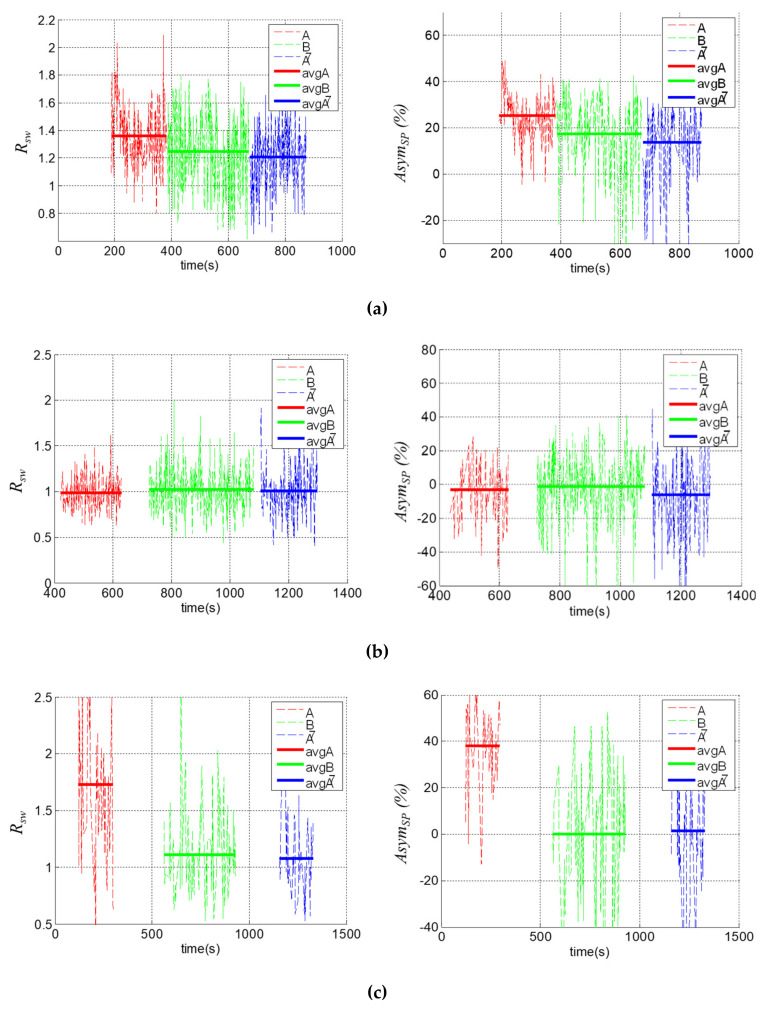

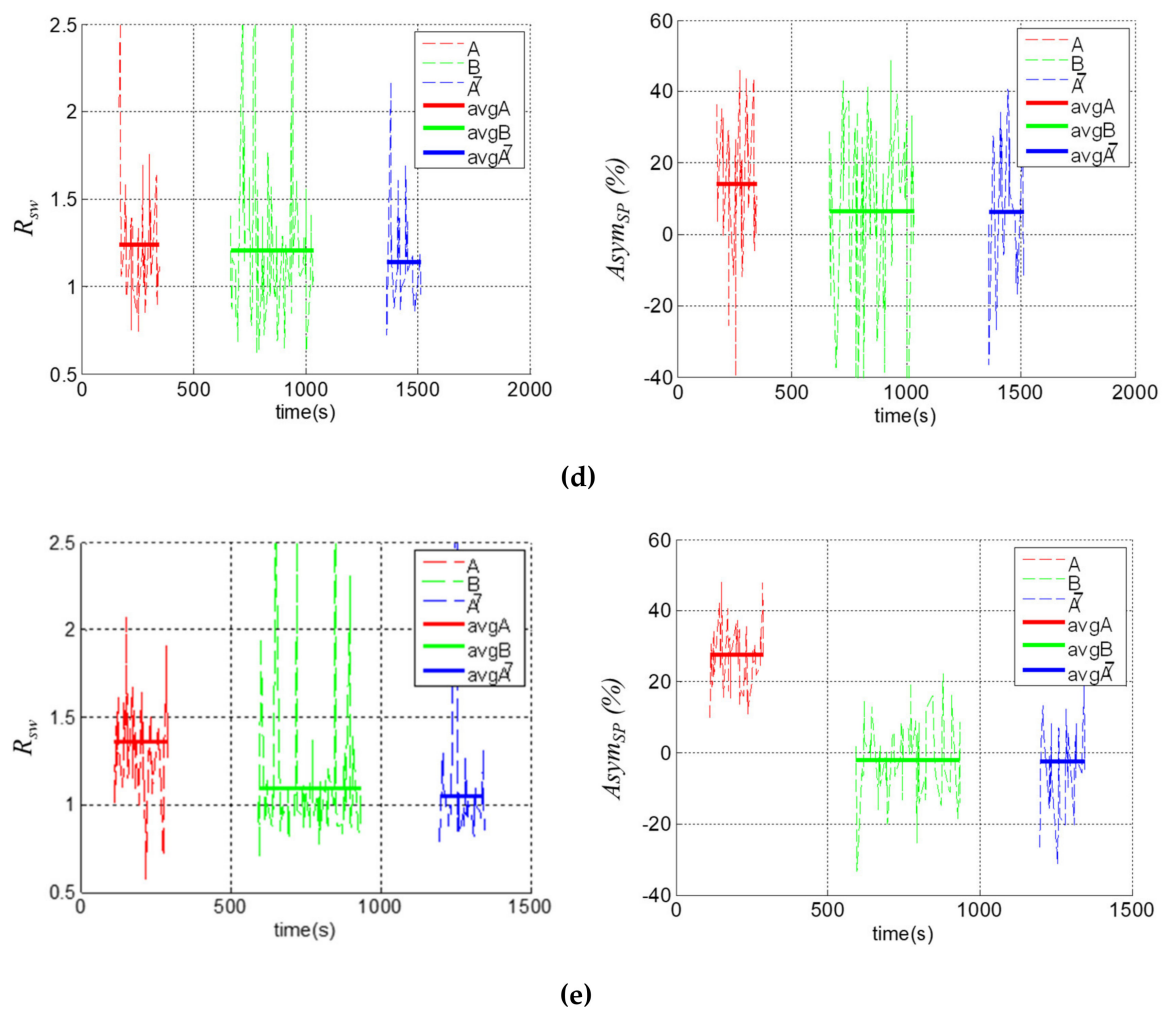
The ratio of the swing time $R_{SW}$ and the asymmetry of the swing phase $Asym_{SP}$. (**a**) For subject P1; (**b**) for subject P2; (**c**) for subject H1; (**d**) for subject H2; (**e**) for subject H3.

We used the two indexes to analyze the testing subjects’ gaits at different stages. The experimental results are shown in Figure 12. First, $R_{SW}$ tended to be a value of one and $Asym_{SP}$ tended to be a value of zero at both the B and $\bar{A}$ stages. That is, the training has positive influences on these subjects and the effects could endure after the treatments. Second, the statistical data of Figure 12 are illustrated in Table 6, where the numbers in bold indicate improvements. Note that $R_{SW}$ almost exceeded a value of one for all subjects because the paretic/restricted side tended to be weak and shortened the swing time on the opposite side. Third, $R_{SW}$ was, in general, improved by the training, except for subject P2, who already had a good recovery in gait symmetry, as indicated by $R_{SW}=0.9880$ at stage A. Therefore, the treatment did not have significant effects on P2. Similarly, $Asym_{SP}$ was generally improved by the training, again except for subject P2, who had already achieved $Asym_{SP}=-3.1273\%$ at stage A. Lastly, the results showed that the automatic trainer had a positive influence on almost all subjects, i.e., their gait symmetry was generally improved by the mobile trainer.

## 6. Conclusions

This paper has demonstrated a mobile trainer designed to help with rehabilitation training of stroke patients. The device consisted of a detection system, a motor control system, and a movable mechanism. First, the detection system was equipped with two measurement units and one data logging unit. The former detected the subjects’ gait data, while the latter transmitted and recorded the data. Gait estimation algorithms were then developed to identify three important gait events: MS, HS, and TO. Second, we conducted clinical NDT training by therapists and recorded the data to describe the therapists’ facilitation patterns. The motor control system was then designed to reconstruct these patterns. Lastly, the detection system and the motor control system were integrated on a movable mechanism, so that the users can move at their own preferred speeds during the training. We conducted experiments and defined two performance indexes to evaluate the effects of the proposed trainer. Based on the results, the automatic trainer was shown to improve the subjects’ walking ability. In the current study, we set strict criteria in recruiting subjects who should be in stable states of stroke and have no other musculoskeletal problems in order to reduce the risk of injuries during the experiments. In the future, we plan to invite more stroke subjects with varieties of gait deficits to participate in more ambulation training to evaluate the long-term rehabilitation effects. 

## Figures and Tables

**Table 1:** System specifications. IMU: inertial movement units.

**IMU (MPU9250) [19,25,26]**	
operating voltage	3.3 V
operating current	3.7 mA
Resolution	16 bits
max measurement range of gyroscope	$\pm {2000\;}^{\circ }/s$
max measurement range of accelerometer	±16 g
max measurement range of magnetometer	±4800 μT
**Wireless transmission module (ESP-01) [21,27]**	
operating voltage	3.3 V
communication protocol	802.11 b/g/n
peripheral interface	UART
working mode	Station/SoftAP/SoftAP+Station
network protocol	TCP/UDP
**Step motor driver (MAC5528) [23]**	
Resolution	500–125,000 steps
max pulse rate	500 kHz
input signal	4–10 V, <20 mA
output signal	24 V, <10 mA
**Step motor (MPK569-2.8A) [23]**	
Phase	5
operating voltage	1.75 V
operating current	2.8 A/phase
static torque	16 kgf⋅cm
**Load cell (MLP-200 [24] & DPM-3 [28,29]**)	
max measurement range of force	200 lb
resonance frequency	5200 Hz
max power consumption	5 W
signal output voltage	0–10 V
signal output current	2 mA
Accuracy	±0.02% of full scale

**Table 2:** Information of the stroke subjects.

Subject	Sex	Age	Height (cm)	Weight (kg)	Paretic Side	MMSE (score)	BS (stage)	FAC (stage)
P1	male	55	155	61	right	30	4	4
P2	male	55	180	75	right	30	4	4

MMSE: Mini-Mental State Examination; BS: Brunnstrom Stage; FAC: Functional Ambulation Category.

**Table 3:** Success rates of the gait detection algorithms.

Subject	Left HS	Right HS
P1	98.4925%	96.4942%
P2	95.4277%	99.2331%

HS: Heel Strike.

**Table 4:** Parameter settings for the motor control system.

	F_max_	F_min_	f(Hz)	Force Command (lb)
Left side	4.9876 lb	0.3739 lb	0.4994	2.30685×sin(2π×0.4994t)+2.68075
Right side	5.8170 lb	0.2223 lb	0.4994	2.79735×sin(2π×0.4994t)+3.01965

**Table 5:** Information of the health subjects.

Subject	Sex	Age	Height (cm)	Weight (kg)	Rehab Gaiter Applied Side
H1	male	24	170	70	right
H2	male	24	176	63	left
H3	male	25	165	60	right

**Table 6:** Performance analyses of Figure 12.

Subject	Index	A	B	$\bar{A}$
P1	$R_{SW}$	1.3604	1.2481	1.2057
	$Asym_{SP}(\%)$	25.1645	17.4078	13.7540
P2	$R_{SW}$	0.9880	1.0187	1.0034
	$Asym_{SP}(\%)$	−3.1273	−0.9489	−6.0481
H1	$R_{SW}$	1.7277	1.1087	1.0753
	$Asym_{SP}(\%)$	38.1344	0.0879	1.3726
H2	$R_{SW}$	1.2410	1.2045	1.1386
	$Asym_{SP}(\%)$	14.0254	6.4010	6.2071
H3	$R_{SW}$	1.3577	1.0928	1.0503
	$Asym_{SP}(\%)$	27.7783	−2.0309	−2.2814

**Figure A1:**
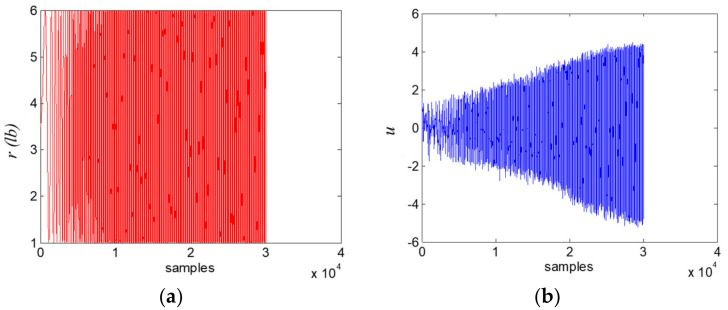

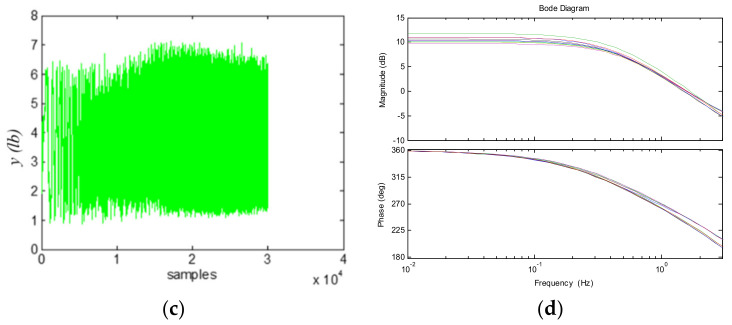
System identification. (**a**) input *r*; (**b**) model input *u*; (**c**) model output *y*; (**d**) Bode plots.

**Figure A2:**
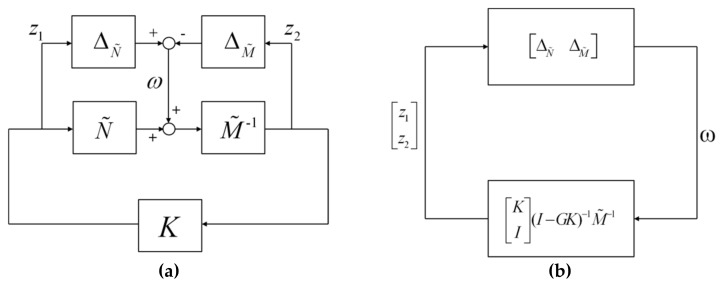
Robust stability analysis. (**a**) closed-loop system with the perturbed plant; (**b**) rearrangement for the Small-Gain theorem.

**Figure A3:**
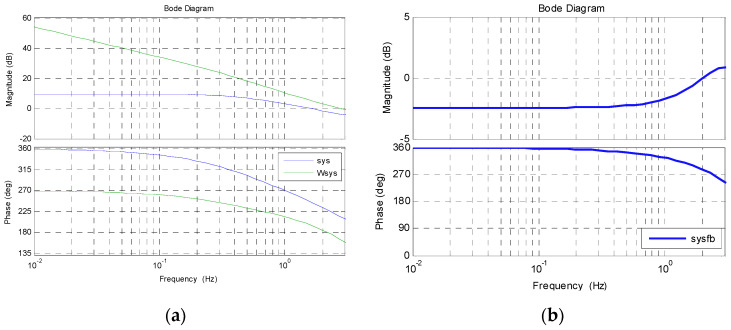
Bode plots for control design. (**a**) Open-loop Bode plots for robust control design; (**b**) Closed-loop Bode plots for pre-compensator design.

## Appendix A. Robust Loop-Shaping Design for the Motor Control System

The motor control system is represented as in Figure 10, where the plant G(s) was obtained by closed-loop identification [39]. We set C(s)=1 and $C_{pre}(s)=1$ and applied a swept sinusoidal signal to the input *r* with a magnitude of 1~6 lb at the frequency range of 0.01–3 Hz. Then we measured *u* and *y* to derive the model $T_{u\to y}$ by the MATLAB command tfest. Considering system uncertainties, we repeated the experiments ten times and obtained the following transfer functions:

(A1) $$G_{i}(s)=T_{u\to y}^{i},\;i=1,2\ldots ,10$$

where $G_{i}$ represented the model derived from the i-th experiment. These signals and the corresponding Bode plots of $G_{i}(s)$ are shown in Figure A1.

From Figure A1d, we can note the model variation, which might be caused by system nonlinearities and operation conditions. Because robust control can cope with system uncertainties, we applied robust control techniques to guarantee system stability and performance. Suppose a nominal plant G with the following left coprime factorization (LCF) [40]:

(A2) $$G={\tilde{M}}^{-1}\tilde{N}$$

where $\tilde{M},\tilde{N}\in RH_{\infty }$ and $\tilde{M}{\tilde{M}}^{*}+\tilde{N}{\tilde{N}}^{*}=I$. Assume a perturbed plant $G_{\Delta }$ can be represented as follows:

(A3) $$G_{\Delta }={\left(\tilde{M}+\Delta_{\tilde{M}}\right)}^{-1}(\tilde{N}+\Delta_{\tilde{N}})$$

where $\Delta_{\tilde{M}},\Delta_{\tilde{N}}\in RH_{\infty }$. The gap between the nominal plant and the perturbed plant can be defined as: the smallest value of ${\left\|\left[\begin{array}{cc} \Delta_{\tilde{N}} & \Delta_{\tilde{M}} \end{array}\right]\right\|}_{\infty }<\varepsilon$ which perturbs G into $G_{\Delta }$ *,* denoted by $\delta (G,G_{\Delta })$. Therefore, we can select the nominal plant from the ten transfer functions by the following equation:

(A4) $$\begin{array}{l} G=\arg\left\{\underset{G}{\min}\;\underset{G_{i}}{\max}\;\delta_{g}\;(G,G_{i})\right\},\;\forall G_{i}\\ =G_{5}(s)=\frac{-16.95s+351.9}{s^{2}+38.9s+114.7} \end{array}$$

which gave $\delta (G,G_{i})\leq 0.1140$. The nominal plant was set to minimize the maximum gaps between plants. The gap can be regarded as the maximum model variation during operations.

The closed-loop system with the controller K and the perturbed plant $G_{\Delta }$ is shown in Figure A2a, which can be arranged as in Figure A2b. From the Small Gain Theorem [41,42], the closed loop system is internally stable for all perturbation with ${\left\|\left[\begin{array}{cc} \Delta_{\tilde{N}} & \Delta_{\tilde{M}} \end{array}\right]\right\|}_{\infty }\leq \varepsilon$ if and only if:

(A5) $${\left\|\left[\begin{array}{c} K\\ I \end{array}\right]{\left(I-GK\right)}^{-1}{\tilde{M}}^{-1}\right\|}_{\infty }={\left\|\left[\begin{array}{c} K\\ I \end{array}\right]{\left(I-GK\right)}^{-1}\left[\begin{array}{cc} I & G \end{array}\right]\right\|}_{\infty }<\frac{1}{\varepsilon }$$

Hence, we can define the stability margin b(G,K) as:

(A6) $$b(G,K)={{\left\|\left[\begin{array}{c} K\\ I \end{array}\right]{\left(I-GK\right)}^{-1}\left[\begin{array}{cc} I & G \end{array}\right]\right\|}_{\infty }}^{-1}$$

so that the system is internally stable for all uncertainty with ${\left\|\left[\begin{array}{cc} \Delta_{\tilde{N}} & \Delta_{\tilde{M}} \end{array}\right]\right\|}_{\infty }\leq \varepsilon$ if and only if b(G,K)>ε.

System performance can be considered by loop-shaping techniques [43,44] with suitable weighting functions, based on the following principles:

(1) increasing system gains at the low frequency for disturbance rejection;
(2) decreasing system gains at high frequency for noise attenuation;
(3) limiting the slope of the magnitude plot less steep than –40 dB/decade around cross-over frequency for stability.

We finally selected the following weighting function by iteratively adjusting the weighting function and verifying the system performance:

(A7) $$W(s)=\frac{1.671s+10.5}{0.03183s^{2}+s}$$

The Bode plot of the shaped plant is shown in Figure A3a. The corresponding robust controller was designed as follows:

(A8) $$K_{\infty }(s)=\frac{-2.801s^{3}-201.5s^{2}-3991s-14390}{s^{3}+103.9s^{2}+7049s+40320}$$

with stability margin $b(WG,K_{\infty })=0.3362$, which was greater than the system gap of 0.1140 and guaranteed system stability during operation. The Bode plots of the plants are shown in Figure A3.

The designed weighted robust controller $K=WK_{\infty }$ was implemented to control the motor system. The Bode plot of the closed-loop system is shown in Figure A3b, where the phase-lag (e.g., $42^{\circ }$ at the frequency of 1 Hz) might cause unsynchronized responses during the training. Therefore, we designed the following phase-lead pre-compensator (see Figure 10) to compensate this phase lag:

(A9) $$C_{pre}(s)=\frac{0.143s+0.4}{0.0709s+1}$$

which gave a phase lead of about $42^{\circ }$ at the frequency of 1 Hz, so that the closed-loop responses will not have phase delay.
